# Lignification of developing maize (*Zea mays* L.) endosperm transfer cells and starchy endosperm cells

**DOI:** 10.3389/fpls.2014.00102

**Published:** 2014-03-20

**Authors:** Sara Rocha, Paulo Monjardino, Duarte Mendonça, Artur da Câmara Machado, Rui Fernandes, Paula Sampaio, Roberto Salema

**Affiliations:** ^1^Departamento de Ciências Agrárias, Instituto de Biotecnologia e Bioengenharia - Centro de Biotecnologia dos Açores, Universidade dos AçoresAngra do Heroísmo, Portugal; ^2^Instituto de Biologia Molecular e Celular, Universidade do PortoPorto, Portugal; ^3^Departamento de Biologia, Faculdade de Ciências, Universidade do PortoPorto, Portugal

**Keywords:** transfer cells, starchy cells, maize endosperm, lignin, flange ingrowths, reticulate ingrowths, cell growth analysis

## Abstract

Endosperm transfer cells in maize have extensive cell wall ingrowths that play a key role in kernel development. Although the incorporation of lignin would support this process, its presence in these structures has not been reported in previous studies. We used potassium permanganate staining combined with transmission electron microscopy – energy dispersive X-ray spectrometry as well as acriflavine staining combined with confocal laser scanning microscopy to determine whether the most basal endosperm transfer cells (MBETCs) contain lignified cell walls, using starchy endosperm cells for comparison. We investigated the lignin content of ultrathin sections of MBETCs treated with hydrogen peroxide. The lignin content of transfer and starchy cell walls was also determined by the acetyl bromide method. Finally, the relationship between cell wall lignification and MBETC growth/flange ingrowth orientation was evaluated. MBETC walls and ingrowths contained lignin throughout the period of cell growth we monitored. The same was true of the starchy cells, but those underwent an even more extensive growth period than the transfer cells. Both the reticulate and flange ingrowths were also lignified early in development. The significance of the lignification of maize endosperm cell walls is discussed in terms of its impact on cell growth and flange ingrowth orientation.

## Introduction

Transfer cells are the first cells to differentiate in maize endosperm and their main purpose is to facilitate the flow of assimilates into the starchy cells. They are characterized by complex flange and reticulate wall ingrowths (Monjardino et al., 2013) that project several micrometers into the cytosol and maintain a consistent ultrastructure throughout development (Davis et al., 1990; Talbot et al., 2002; Offler et al., 2003; McCurdy et al., 2008; Monjardino et al., 2013). The starchy cells are the most abundant cells in the endosperm. They predominantly accumulate starch and protein starting 12–14 days after pollination (DAP) and continuing until physiological maturity (Young and Gallie, 2000; Monjardino et al., 2005; Prioul et al., 2008). They undergo programed cell death during endosperm development (Young and Gallie, 2000), but nevertheless withstand severe desiccation and maintain their tightly-arranged starch granules and protein bodies.

Lignification is associated with several traits in plants, including the structural integrity of the cell wall, secondary growth of vascular tissues, the strength of the stem and root (Boerjan et al., 2003), resistance to fungi (Ride, 1975; Xu et al., 2011), the prevention of autolytic cell wall degradation (O'Brien, 1970), and the gravitropic response of trees (Donaldson et al., 2010). Lignins are the second most abundant polymers in plants (after cellulose) and comprise phenolic heteropolymers resulting from the oxidative coupling of the three *p*-hydroxycinnamyl alcohols: *p-coumaryl*, coniferyl and sinapyl (Lewis and Yamamoto, 1990; Dixon et al., 2001). The polymerization of *p*-hydroxyphenyl, guaiacyl, and syringyl monomers via carbon–carbon and ether linkages is mediated by free radicals (Nadji et al., 2009; Liu, 2012; Zhang et al., 2012).

Lignin has been suggested to be present in the ingrowths of transfer cells adjacent to sieve elements of basal nodes of the perennial *Valeriana officinalis* plants (Gaymann and Lörcher, 1990), using toluidine blue stain to detect it. In addition Heide-Jørgensen and Kuijt (1994) have detected lignin in transfer cells situated between the root xylem elements of *Triphysaria* sp. plants and their hosts with phloroglucinol. However, other studies using the periodic-Schiff reaction with alcian blue or with toluidine stains failed to detect lignification in transfer cells compared to xylem cells in the nodes of *Trifolium repens* and *Trollius europaeus* (Gunning and Pate, 1974). Furthermore, lignin was not detected with phloroglucinol in *Vicia faba* cotyledon transfer cells (Vaughn et al., 2007), which have led to a general consensus that transfer cells are not lignified (Offler et al., 2003; McCurdy et al., 2008). However, these methods may not be sensitive enough to detect small amounts of lignin, e.g., phloroglucinol does not detect the early stages of lignification and a negative phloroglucinol reaction therefore does not necessarily confirm the absence of lignin (Kutscha and McOrmond, 1972; Müsel et al., 1997). It has been demonstrated that ferulic and p-coumaric acids, two precursors of lignin, can esterify to lignin and to polysaccharides of the wall of the *Poaceae* (Harris and Hartley, 1976), including in tissues that give a negative phloroglucinol reaction. It is possible that may be the case in the endosperm transfer cells.

Potassium permanganate (KMnO_4_) is a general electron-dense staining agent for lignin, which works by oxidizing coniferyl groups. The permanganate anion is reduced to insoluble manganese dioxide (MnO_2_) which then precipitates, indicating the reaction site (Hepler et al., 1970; Bland et al., 1971; Kutscha and Gray, 1972; Xu et al., 2006; Ma et al., 2011). Ultrathin sections can be stained with KMnO_4_ to determine the distribution of lignin in woody cell walls (Donaldson, 1992; Grünwal et al., 2002; Coleman et al., 2004; Wi et al., 2005; Xu et al., 2006; Lee et al., 2007; Tao et al., 2009; Ma et al., 2011).

Scanning electron microscopy and transmission electron microscopy (TEM) can be used to generate backscattered electrons for energy dispersive X-ray spectrometry (EDS), and these techniques can be used to probe the results of KMnO_4_ staining (Stein et al., 1992; Xu et al., 2006; Ma et al., 2011). The greater the concentration of Mn revealed by TEM–EDS, the higher the lignin concentration (Xu et al., 2006), and these data can be used for the quantitative assessment of lignin distribution (Ma et al., 2011). Bland et al. (1971) and Hoffmann and Parameswaran (1976) found that KMnO_4_ can stain several amino acids and other cell wall components with acidic groups in addition to lignin, but their studies involved chemically-delignified plant material and acidic groups that are rare in native plant cell walls. However, fixatives such as glutaraldehyde can react with the aminoacids lysine, tyrosine, tryptophan, phenylalanine, hystidine, cysteine, proline, serine, glycine, glycilglycine, and arginine (Migneault et al., 2004), therefore their reactivity to KMnO_4_ may be altered in fixed tissues. Coleman et al. (2004) highlighted the duration of KMnO_4_ staining, because excessive exposure can result in non-specific staining of the cell wall based on the potent oxidation activity of this chemical (Lawn, 1960).

Acriflavine is a fluorochrome that can detect low levels of lignin. The intensity of acriflavine fluorescence is proportional to the concentration of lignin, and the signal can be detected and quantified by confocal laser scanning microscopy (CLSM) (Donaldson et al., 2001; Coleman et al., 2004; Christiernin et al., 2005; Cho et al., 2008; Nakagawa et al., 2012) or epifluorescence microscopy (Donaldson and Bond, 2005).

Lignin can be extracted using solvents containing hydrogen peroxide (H_2_O_2_), so the same method can be used for lignin detection (Xiang and Lee, 2000; Svitelska et al., 2004; Yao et al., 2006; Hejri and Saboora, 2009). This technique is often more useful when applied in combination with other detection strategies.

There are several methods that chemically extract and detect lignin from plant tissues that vary in sensitivity and specificity (Hatfield and Fukushima, 2005). Each methodology carries its specific constrains and one that is relevant to this study is the rather limited amount of tissue to be analyzed. The transfer cells are located in the placento-chalazal region of the endosperm, their tissue often has a total volume of ~0.1 mm^3^, therefore even when large numbers of samples are pooled it may not be enough for most of the methods for lignin analysis. The acetyl bromide method combines the specificity, the sensitivity and the relatively low requirement of plant tissue (Fukushima and Hatfield, 2001; Foster et al., 2010).

We determined the presence of lignin in developing transfer cells and starchy cells using two staining techniques: KMnO_4_ in conjunction with TEM–EDS and acriflavine in conjunction with CLSM. We also studied H_2_O_2_-treated samples by TEM and TEM–EDS. The lignin content of the walls was also determined by acetyl bromide method, we analyzed the growth of both types of cells, and determined the flange ingrowth orientation in transfer cells. Our results revealed that transfer cells become lignified during early development when undergoing active growth and the formation of cell wall ingrowths. Similar levels of lignification were observed in the reticulate and flange ingrowths and cell walls at mid-development stages. The cell walls of starchy cells also became lignified from early through late developmental stages.

## Materials and methods

### Plant material, growth conditions, and sampling

Maize plants (inbred W64A) were cultivated and kernels were collected from 2009 to 2013 as previously described (Monjardino et al., 2013). At each sampling date, at least 30 kernels were collected from at least five different ears. We focused on the most basal endosperm transfer cells (MBETCs) and the starchy cells.

The temperature was recorded daily during early kernel development allowing the growing degree days (GDD) to be calculated according to the formula GDD = Σ (ADT − BT), where ADT is the average daily temperature and BT is the base temperature of 10°C (Gilmore and Rogers, 1958). Minimum temperatures <10°C were adjusted to 10°C, and maximum temperatures >30°C were adjusted to 30°C. The developmental stages were therefore described as DAP and references were made to GDD.

### TEM–EDS of samples stained with KMnO_4_

Kernels were selected at 6, 12, and 20 DAP (95–97.5, 190–192.5, and 315.5–318.5 GDD, respectively), and were sectioned with a razor blade, discarding most of the endosperm tissue except the basal and central endosperm regions. The tissues were fixed immediately in 4% glutaraldehyde containing 2% osmium tetroxide for 2 h. The fixed sections were dehydrated in acetone and progressively infiltrated in Spurr's resin for 8 days at room temperature (Monjardino et al., 2007) before polymerization at 60°C.

**Figure 1:**
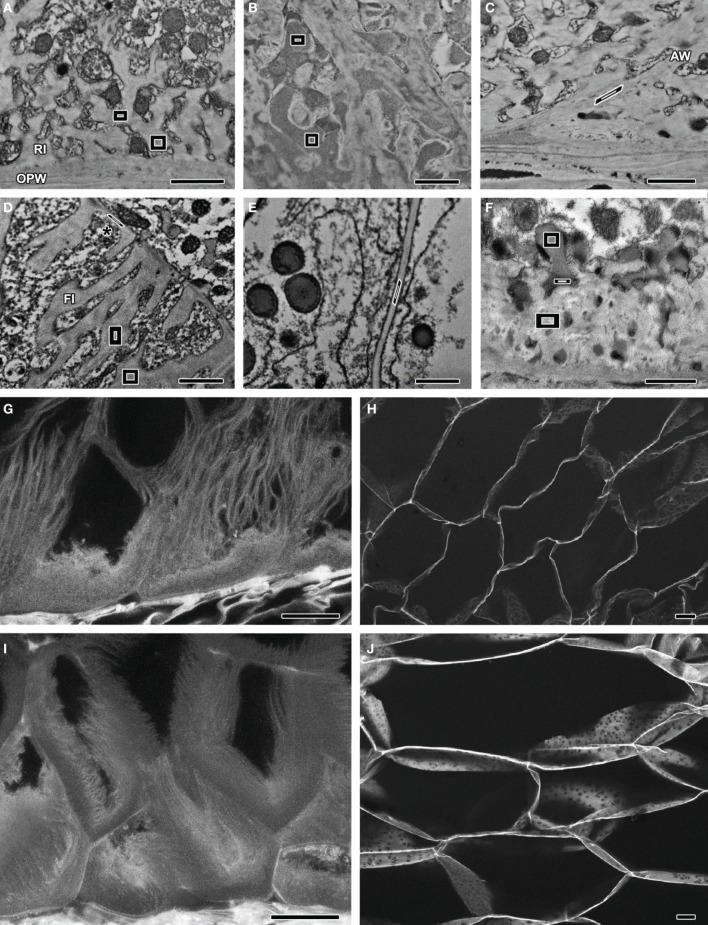
**Images obtained by TEM-EDS **(A–E)** and TEM **(F)** of samples stained with KMnO_4_.** Polygons represent the boundaries of EDS readings in each sample. **(G–J)** CLSM images of acriflavine-stained samples. **(A)** MBETCs at 12 DAP – readings were obtained from the reticulate ingrowths. **(B)** MBETCs at 12 DAP – readings were obtained from vesicles adjacent to reticulate ingrowths. **(C)** MBETCs at 12 DAP – readings were obtained from anticlinal walls. **(D)** MBETCs at 20 DAP – readings were obtained from flange ingrowths and inner periclinal walls (^*^). **(E)** Starchy cells at 20 DAP – readings were obtained from the walls. **(F)** MBETCs at 6 DAP – readings were obtained from vesicles releasing their contents into reticulate ingrowths. **(G)** MBETCs at 9 DAP. **(H)** Starchy cells at 9 DAP. **(I)** MBETCs at 25 DAP. **(J)** Starchy cells at 25 DAP. *AW*, anticlinal wall; *FI*, flange ingrowth; *OPW*, outer periclinal wall; *RI*, reticulate ingrowth. *Scale bars*: **(A–F)** = 1 μm; **(G–J)** = 20 μm.

Ultrathin sections (40–60 nm) were prepared on a LKB 2188 NOVA Ultramicrotome (LKB NOVA) using diamond knives (Diatome). The sections were mounted on 400-mesh gold grids (Agar Scientific), stained with 1% KMnO_4_ for 2 min each, followed by eight washes in water, and examined under a JEOL JEM 1400 TEM equipped with an EDS Microanalysis System (Oxford Instruments). We analyzed MBETC and starchy cell walls, ingrowths and vesicles adjacent to the reticulate ingrowths (when visible). The areas were traced for the probe to raster and produce an integrated average of several thousand readings (Figures 1A–F). Areas of the sample that apparently did not contain any cell content were used as controls. The elements analyzed in the samples were C, O, Mn, Na, K, Ca, and Fe. The levels of each element were expressed in terms of the relative proportion in relation to the sum of the seven elements.

### TEM and TEM–EDS following exposure to H_2_O_2_

Kernels were selected at 5–20 DAP (69.5–77.5 to 284.0–287.5 GDD) and processed as described above. After mounting on 400-mesh gold grids, the ultrathin sections were treated with 4% H_2_O_2_ for 15 or 60 min, followed by three washes in water. Images were captured using a Zeiss EM10 C TEM (Carl Zeiss) and were digitally recorded using a Gatan SC 1000 ORIUS CCD camera (Warrendale).

Ultrathin sections containing MBETCs of 6 DAP and 12 DAP kernels (97.5 and 191 GDD, respectively), were treated with 3% H_2_O_2_ for 60 min, followed by eight washes in water, after which they were stained with 1% KMnO_4_ for 2 min, followed by three washes in water, and examined under a JEOL JEM 1400 TEM equipped with an EDS Microanalysis System. The element analysis was conducted as described above. The percentage reduction of Mn deposition due to H_2_O_2_ treatment was calculated by comparison to other ultrathin sections of the same samples that were only stained with 1% KMnO_4_ for 2 min.

### CLSM with acriflavine staining

Kernels were selected at 6, 9, and 25 DAP (92.0–92.5, 128–134.5, and 355.5–356.5 GDD, respectively), and sectioned longitudinally (70–100 μm thicknesses) with a Leica VT 1200 vibratome (Leica Microsystems). The sections were stained with 0.0025% aqueous acriflavine for 10 min, followed by a 2-min wash in water, and coverslips were mounted with glycerol (adapted from Donaldson and Bond, 2005). Unstained sections were used as controls. The sections were visualized under a Zeiss CLSM 510 (Carl Zeiss) at an excitation wavelength of 488 nm and emission wavelengths of 505–550 nm and LP 615 nm for detection. The projected images were obtained from Z stacks at a resolution of 1024 × 1024 pixels. The Z stacks contained 16–35 planes at 0.37-μm intervals.

### Lignin determination by acetyl bromide method

Kernels of 5, 10, and 15 DAP (61.5–77.5, 140–152.5, and 211–231 GDD, respectively), were dissected manually and the transfer cell and starchy cell tissues were collected separately, frozen in liquid nitrogen and stored at −80°C until further use. There was a particular concern in dissecting the transfer cell tissue in order to not include remnants of the nucellus, the pedicel and pericarp, and the embryo tissue. The starchy cells were collected from the central endosperm region. For each sampling at least 120 kernels were dissected and at least four samples were pooled in each replicate in order to have a minimum of 50 mg of dry matter for the extraction of wall material (Foster et al., 2010). The extraction of lignin was done according to the procedures of Fukushima and Hatfield (2001) and absorbance was measured at 280 nm by a double beam UV-Vis spectrophotometer Shimadzu UV-2401 PC (Shimadzu Corporation). The analysis was done on two replicates of transfer cells and starchy cells of 10 and 15 DAP kernels and on the pedicel and pericarp of 5 DAP kernels. Stems of *Populus nigra* L. were used as control. The percentage of acetyl bromide soluble lignin was determined using an appropriate coefficient (maize = 17.75 L g^−1^ cm^−1^; *Populus nigra* = 18.21 L g^−1^ cm^−1^) with the formula described in Foster et al. (2010).

### Analysis of growth and flange ingrowth angle

For growth analysis, kernels at 4–35 DAP (62.0–65.5 to 515.5–522.0 GDD) were processed as described above for CLSM, but were stained with 0.01% calcofluor white (Monjardino et al., 2013). For flange ingrowth angle analysis, kernels at 6, 12, and 20 DAP (83.5–93, 168.0–177.5, and 284.0–287.5 GDD, respectively), were processed in the same manner. Sections were visualized under a Zeiss CLSM 510 at an excitation wavelength of 405 nm (UV diode laser) and an emission wavelength of 420–480 nm. The projected images were obtained from Z stacks at a resolution of 1024 × 1024 pixels. The Z stacks contained 25–98 planes at 0.37-μm intervals.

Cell areas (taken from two-dimensional CLSM images) were measured from an average of seven cells per kernel and 15 kernels per sampling date. Flange ingrowth angles were measured from an average of three cells per kernel and five kernels per sampling date. Cell areas were measured from contiguous MBETCs with Zeiss software LSM 510 4.0 SP1 using the “closed free-shape curve” feature from the “overlay toolbar.” Flange ingrowth angles in MBETCs were measured relative to the nearest anticlinal wall using the “line” feature from the “overlay toolbar” in the same software.

All selected images were imported into Adobe Photoshop CS6 software (Adobe Systems) for presentation, and photomontages were produced using the same software.

### Statistical analysis

A complete randomized model was used to analyze the TEM–EDS data, and ANOVA was carried out using Microsoft Excel. Means were compared using the Tukey multiple range test. For growth analysis, a regression survey was conducted using CurveExpert Professional software with GDD as the independent variable and cell area as the dependent variable. Among several tested models, the Richards sigmoidal function gave the best fit for MBETC growth analysis, whereas the logarithmic function gave the best fit for starchy cell growth analysis.

## Results

### TEM–EDS analysis

Our TEM analysis highlighted some differences between reticulate ingrowths, vesicle content, anticlinal walls, flange ingrowths, inner periclinal walls, and starchy cell walls, but without sufficient clarity to distinguish them (Figures 1A–F). However, the additional EDS quantification mode allowed us to measure the amount of Mn and thus to infer the pattern of lignin deposition. Manganese could be detected as early as 6 DAP, mainly in the reticulate ingrowths (Figure 1A) and vesicles (Figure 1B), and also to a lesser extent in the anticlinal walls (Figure 1C), the flange ingrowths and inner periclinal walls (Figure 1D), and starchy cell walls (Figure 1E, Table 1). The reticulate ingrowths and vesicles contained significantly higher levels of Mn than the flange ingrowths, the anticlinal walls and periclinal walls at 6 DAP, whereas at 12 DAP the reticulate ingrowths, vesicles and anticlinal walls had significantly higher levels of Mn than the flange ingrowths, periclinal walls and starchy cell walls. At 20 DAP, there were no significant differences in Mn levels among the vesicles, ingrowths, and walls of the MBETCs and starchy cells. The Mn levels in the periclinal walls increased significantly throughout development, whereas the other cellular components did not show substantial variation (Table 1).

**Table 1:** **The average percentage concentration of elemental Mn in transfer cells and starchy cells at 6, 12, and 20 DAP, determined by TEM–EDS**.

	**6 DAP**	**12 DAP**	**20 DAP**	***P***
Reticulate ingrowths	8.18 a^1^, A^2^	7.43 a^1^, A	7.84 a^1^, A	>0.05
Vesicles adjacent to reticulate ingrowths	9.35 a, A^2^	8.66 a, A	10.25 a, A	>0.05
Anticlinal walls next to the OPV^§^	6.00 a, B^2^	7.61 a, A	7.05 a, A	>0.05
Flange ingrowths	6.26 a, B^2^	5.53 a, B	6.48 a, A	>0.05
Periclinal walls	5.50 b, B^2^	4.98 b, B	7.69 a, A	0.02
Starchy cell walls	–	3.35 a, C	5.68 a, A	>0.05
Control	0.63 a, C^2^	0.47 a, D	0.56 a, B	>0.05
*p*	7.94 × 10^−5^	1.12 × 10^−6^	4.45 × 10^−3^	

1 Numbers in lines followed by the same lower case letter do not differ significantly.

2 Numbers in columns followed by the same capital letter do not differ significantly.

§ OPW, outer periclinal wall.

Vesicles often fused with reticulate ingrowths and released their highly electron-dense contents (Figure 1F). TEM–EDS analysis revealed a fusing vesicle containing 9.10% Mn immediately adjacent to an electron-dense region in the reticulate ingrowths containing 8.27% Mn, whereas ~500 nm further away the reticulate ingrowths contained only 7.62% Mn (Figure 1F). This suggests that a substantial portion of lignin or its precursors must be transported through the vesicles into the reticulate ingrowths.

### CLSM analysis of acriflavine-stained samples

The fluorescence of acriflavine was only detected in the 505–550 nm band, not above 615 nm, which suggests that it specifically detected lignin (Donaldson et al., 2001). Sections of maize kernels stained with acriflavine revealed weak fluorescence in the MBETCs at 6 DAP, except for the region next to the outer periclinal wall (OPW) where the fluorescence was more intense, with similar staining in the future starchy cells (data not shown). These data suggested that there is only a low level of lignification in the endosperm cell walls at this stage. Later in development (9 DAP), the fluorescence levels increased in the transfer cells (Figure 1G) and starchy cells (Figure 1H). This represented the mid-phase of ingrowth development in the transfer cells (Monjardino et al., 2013), whereas starch and protein accumulation was about to begin in the starchy endosperm cells. At 25 DAP, high-intensity acriflavine labeling was observed in both the transfer cell walls and their ingrowths (Figure 1I). At this stage, the walls of the MBETCs showed more intense fluorescence than the adjacent ingrowths, suggesting a greater degree of lignification. The starchy cell walls were also more intensely fluorescent at this stage (Figure 1J), suggesting that lignification increased throughout development. These data supported the TEM-EDS results. Lignification could be detected from early developmental stages using both techniques. At 9 DAP, the walls and ingrowths of the transfer cells were more intensely stained than the starchy cell walls, but by 25 DAP the MBETC and starchy cell walls stained with similar intensity suggesting they contained equivalent amounts of lignin.

### TEM analysis of H_2_O_2_-treated samples

**Figure 2:**
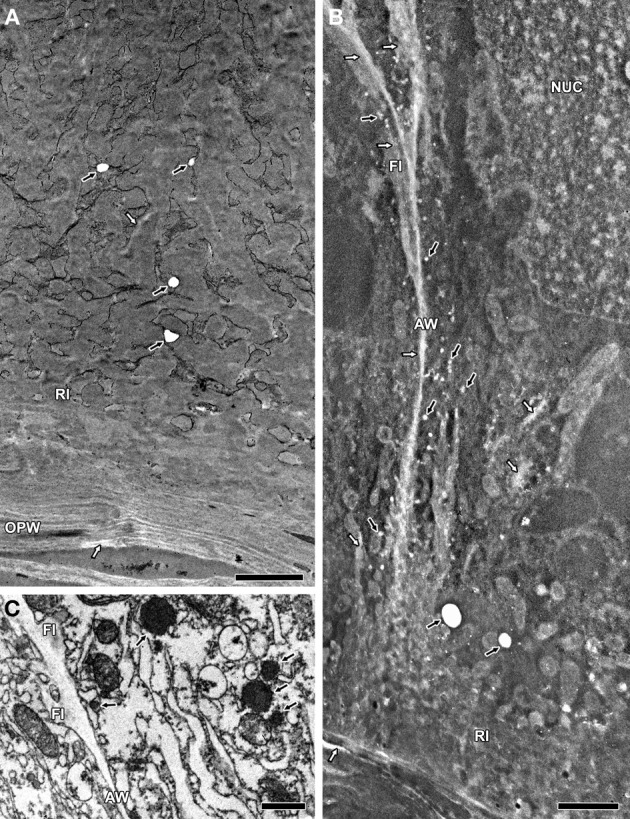
**TEM images of ultrathin MBETC sections at 10 DAP, after H_2_O_2_ treatment without contrast (A and B) and control sections at 6 DAP without treatment and contrast (C). (A)** After a 15-min H_2_O_2_ treatment, some vesicles lacked electron density (*black arrows*), whereas reticulate ingrowths and the OPW show regions with less electron density (*white arrows*). **(B)** After a 60-min H_2_O_2_ treatment, many vesicles lost their electron density (*black arrows*), whereas reticulate and flange ingrowths, the OPW and anticlinal walls reveal large regions with less electron density (*white arrows*). **(C)** Control sections with electron-dense vesicles (*black arrows*), whereas the walls and flange ingrowths have a weak electron density. *AW*, anticlinal wall; *FI*, flange ingrowth; *NUC*, nucleus; *OPW*, outer periclinal wall; *RI*, reticulate ingrowth. *Scale bars*: **(A,B)** = 2 μm, **(C)** = 1 μm.

Ultrathin sections of differentiating transfer cells were treated with H_2_O_2_ and analyzed by TEM, revealing that some vesicles lacked their electron-dense contents (Figures 2A,B). Because these samples were not stained prior to analysis, the observed electron density thus arose from their intrinsic characteristics, the osmium fixation and the treatment with H_2_O_2_. The visualization of untreated controls of the same samples showed that similar vesicles were electron dense (Figure 2C). This indicated that the loss of electron density was a direct consequence of the H_2_O_2_ treatment, and strongly suggested the presence of polyphenolic compounds in the vesicles.

The lack of electron density was affected by the duration of H_2_O_2_ treatment. After 15 min exposure, only part of the vesicles and a few scattered regions of the reticulate ingrowths and OPW lacked the electron density (Figure 2A). However, after 60 min exposure there was more extensive loss of electron density in the vesicles and partial or complete loss in the walls and ingrowths (Figure 2B). These data also supported the presence of polyphenolic compounds in the vesicles, walls and ingrowths of MBETCs.

### TEM–EDS analysis of H_2_O_2_-treated samples

The H_2_O_2_ treatment prior to KMnO_4_ staining caused drastic reductions of the Mn levels in the vesicles, ingrowths and walls of 6 and 12 DAP MBETCs (Table 2). The vesicles suffered the largest reduction in Mn levels, followed by the reticulate ingrowths, the walls and the flange ingrowths.

**Table 2:** **The average percentage reduction of Mn deposition after H_2_O_2_ treatment prior to KMnO_4_ staining in MBETC at 6 and 12 DAP, determined by TEM–EDS**.

	**6 DAP**	**12 DAP**
Reticulate ingrowths	58.56	54.22
Vesicles adjacent to ingrowths	77.66	60.24
Flange ingrowths	48.77	43.98
Walls	47.96	48.15

### Lignin determination by acetyl bromide method

The lignin levels in the wall extracts of transfer and starchy cells of 10 and 15 DAP kernels were relatively constant, ranging between 36.9 and 47.4 μg mg^−1^ (Table 3). Although the extraction of the wall material involves steps that should remove the precursors of lignin, we aren't sure on whether they were totally removed, therefore they could have been extracted together with lignin by acetyl bromide. The small variability of lignin content between these tissues is similar to the TEM–EDS analysis, but the slight tendency of the lignin levels to decrease from 10 to 15 DAP is opposite to the TEM–EDS analysis. The pedicel and pericarp of 5 DAP kernels have a much higher lignin content, which is expected, because the vascular bundles had a positive phloroglucinol reaction, although the parenchyma cells had a negative reaction (data not shown). The *Populus nigra* wood sample had a lignin concentration of 199.2 μg mg^−1^, which is similar to previously published data using the same extraction and detection methods (Foster et al., 2010).

**Table 3:** **Average lignin content (±standard error) in the dry matter of wall material (μg mg^−1^) extracted by the acetyl bromide method (Fukushima and Hatfield, 2001; Foster et al., 2010)**.

	**Lignin content**
*Populus nigra* wood	199.2
Pericarp and pedicel 5 DAP	80.5 ± 13.2
Transfer cells 10 DAP	46.4 ± 14.8
Starchy cells 10 DAP	47.9 ± 9.7
Transfer cells 15 DAP	38.0 ± 10.0
Starchy cells 15 DAP	36.9 ± 12.4

### Analysis of cell growth and flange ingrowth angle

**Figure 3:**
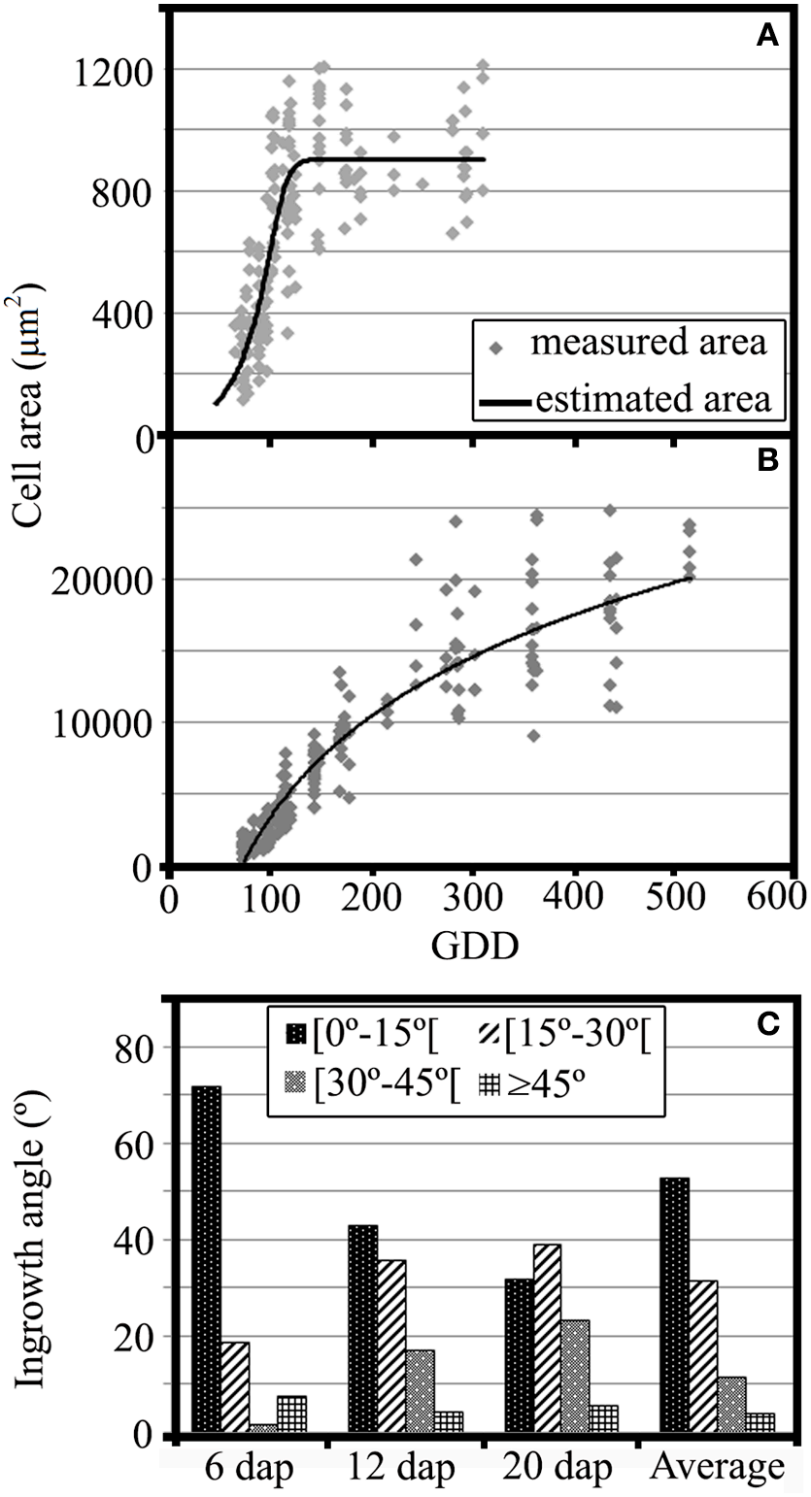
**(A)** Growth analysis of MBETCs, showing cell areas (μm^2^) of developing kernels (4–20 DAP, equivalent to 62.0–300.2 GDD) and adjusted growth curve (cell area = 903.04/(1 + exp(15.81 − 0.15 × GDD)^∧^(1/4.37)), *R*^2^ = 67.15%). **(B)** Growth analysis of starchy cells, showing cell areas (μm^2^) of developing kernels (5–35 DAP, equivalent to 73.5–522.0 GDD) and adjusted growth curve (cell area = −43582.6 + 10195.3 × ln(GDD), *R*^2^ = 92.3%). **(C)** Flange ingrowth angles (in relation to the nearest anticlinal wall) of developing MBETCs (6, 12, and 20 DAP, and an average of the three sampling dates).

The transfer cells expanded until 120–140 GDD (9–10 DAP; Figure 3A) whereas the starchy cells were still expanding at 520 GDD (35 DAP; Figure 3B). The transfer cells reached their maximum growth rate at 96 GDD (6 DAP; Figure 3A), when the amount of lignin was relatively low in all structures except the reticulate ingrowths and vesicles (Table 1). The starchy cells also showed their highest growth rates prior to 100 GDD, but the subsequent reduction in growth rate was not as dramatic as observed for the transfer cells (Figure 3B). Indeed, lignification coincided with starchy cell growth throughout development, which clearly shows that lignification does not inhibit the growth of these cells. The starchy cells continued expanding intensively at 35 DAP, probably reflecting the active accumulation of assimilates at this stage.

The flange ingrowth angles varied dramatically throughout development (Figure 3C). At 6 DAP, most of the flange ingrowths had angles of less than 15° relative to the nearest anticlinal wall, whereas later there was a greater proportion of angles between 15 and 30°. Furthermore, the proportion of angles between 30 and 45° increased as the MBETCs developed. The proportion of flange ingrowths angles greater than 45° remained at <10% throughout development, which confirmed previous observations that such ingrowths tend to be oriented longitudinally and obliquely, rather than transversely to the longer axis of the cell (Monjardino et al., 2013).

## Discussion

Our analysis revealed that transfer cells have extensive inward wall projections that contrast with the much thinner walls of the starchy cells (Figure 1). The inward growth of cell wall regions must overcome the outward pressure of the living cell cytoplasm, and it is not unreasonable to suggest that this process might require reinforcement, e.g., by the ubiquitous structural molecule lignin.

The TEM–EDS technique allowed the accurate quantification of elemental Mn deposited in the cell walls and their ingrowths. Our data were based on the trusted and reliable Clifft Lorimer thin ratio section quantification method, which revealed that Mn was deposited on the walls of the transfer cells and starchy cells (Table 1, Figures 1A–F). Because TEM–EDS elemental analysis offers a high degree of confidence, it was possible to conclude that Mn atoms were deposited in the walls and ingrowths in amounts that could not be attributed to background counts observed in the control sections (Table 1). Considering previous reports that KMnO_4_ does not react with cellulose, hemicelluloses or pectin (Kutscha and Gray, 1972) and that short exposure times prevent non-specific staining (Coleman et al., 2004), it would be reasonable to conclude that the KMnO_4_ reacted with lignin, leading to the deposition of Mn atoms. However, it cannot be ruled out the reaction of KMnO_4_ with other wall constituents, namely precursors of lignin like the ferulic and p-coumaric acids, which have been demonstrated to exist in relatively high content in the cell walls of *Poaceae* plants, as compared to lignin (Harris and Hartley, 1976). It is also possible that KMnO_4_ may react with some amino acids (Bland et al., 1971) and acidic groups (Hoffmann and Parameswaran, 1976) like galacturonic and glucuronic acids. Therefore, we consider that the KMnO_4_ staining and TEM-EDS analysis must have provided a proportional indication of the lignin content, but not the exclusive proof of its existence.

H_2_O_2_ treatment provided an indirect approach to confirm the presence of lignin: short-exposure experiments (15 min; Figure 2A) resulted in the loss of electron-dense material from vesicles compared with untreated control samples (Figure 2C), whereas longer exposure (60 min) also caused the loss of electron-dense material from ingrowths and walls (Figure 2B). These results suggest that vesicles may contain the highest concentrations of polyphenolic compounds in the MBETCs but also that ingrowths and walls must have lower but still biologically-relevant concentrations of such constituents. The presence of vesicles that support ingrowth formation has been reported previously (Davis et al., 1990; our unpublished data). The vesicles are numerous (Figure 2B) and they contribute to ingrowth formation (Figure 1F), thus reinforcing the thesis of exocytosis supporting lignin deposition in the ingrowths.

We also tested the effects of H_2_O_2_ treatment prior to KMnO_4_ staining and quantified it by TEM–EDS in the MBETCs (Table 2). The reduction of Mn deposition was the highest in the vesicles at 6 and 12 DAP, thus suggesting to contain the highest lignin and/or its precursors' content. The reticulate ingrowths had the second highest reduction of Mn deposition due to H_2_O_2_ treatment, which is also in accordance with the TEM–EDS data (Table 1). The flange ingrowths and walls had the lowest reduction of Mn deposition, but it still was above 40%, which reinforces the concept of them also being lignified, although at lower levels than the reticulate ingrowths.

The acriflavine staining experiment confirmed the presence of lignin in the transfer cell walls and ingrowths, as well as in the walls of the starchy cells (Figures 1G–J). The intensity of the staining mirrored the TEM–EDS data, thus reinforcing our conclusions. It should be highlighted the tendency of the inner edges of the reticulate ingrowths to fluoresce more intensively than the rest of these ingrowths.

The determination of lignin content by the acetyl bromide method (Fukushima and Hatfield, 2001; Foster et al., 2010) was essential to confirm the presence of lignin and its precursors in the walls of the transfer and starchy cells of maize endosperm (Table 3), regardless of the negative phloroglucinol reaction. However, this methodology does not enable us to distinguish the ingrowths from the adjacent walls. The slight tendency of reduction of the lignin and its precursors content from 10 to 15 DAP (Table 3) is not fully in accordance to the TEM–EDS data (Table 1) and that could be due to: (a) non-specific determination of other wall and ingrowth components by TEM–EDS that would partially mask the lignin determination; (b) the extraction of wall material prior to the acetyl bromide analysis not being 100% efficient and still remnants of starch and other cell components of 15 DAP kernels causing an unforeseeable dilution of lignin. This analysis clearly makes the case that lignin exists in the transfer and starchy cell walls throughout kernel development.

Consistently higher levels of Mn were deposited in reticulate ingrowths compared to flange ingrowths, suggesting that larger amounts of lignin may be required to stabilize the former structures. Reticulate ingrowths contain less-compacted cellulose microfibrils that tend to be arranged in non-parallel arrays (Offler et al., 2003; McCurdy et al., 2008; Monjardino et al., 2013), whereas the cellulose microfibrils in flange ingrowths are arranged in tight parallel arrays. Therefore, it seems plausible that higher lignin levels may be required to stabilize the reticulate ingrowths because of their unique ultrastructure. The stronger fluorescence of acriflavine in the inner edges of the reticulate ingrowths makes stronger the assumption of lignin to be determinant to stabilize the looser ingrowth structures. To our knowledge this trait has not been documented before and it makes a strong case for the relevance of lignin to ingrowth formation.

The presence of high Mn levels in vesicles adjacent to the reticulate ingrowths (Figures 1B,F, Table 1) and the most drastic reduction of Mn deposition after H_2_O_2_ treatment (Table 2) support the exocytosis of lignin or its precursors in vesicles derived from the Golgi body (reviewed by Donaldson, 2001). In the transfer cells, the flux of vesicles apparently from the Golgi body is intense throughout development (Davis et al., 1990; Monjardino et al., 2013) and this offers a significant mechanism to transport lignin into the ingrowths and adjacent cell walls, although other mechanisms cannot be ruled out (Liu, 2012; Wang et al., 2013). These results point to the need of using other methods to assess the transportation role of Golgi vesicles before definite conclusions could be drawn.

Basic fuchsin in combination with fluorescence or light microscopy has also been used successfully as a staining method for lignified cell walls (Fuchs, 1963; Dharmawardhana et al., 1992; Kraus et al., 1998; Caño-Delgado et al., 2000; Möller et al., 2005; Soyano et al., 2008; Wagner et al., 2009). In a previous study, we showed that basic fuchsin reacts with ingrowths and adjacent walls (Machado, 2004). However, other reports indicated that basic fuchsin has affinity for the suberized and cutinized walls of plants cells and other structures devoid of lignin, such as chloroplasts, and starch granules in maize endosperm (Kraus et al., 1998; Machado, 2004; Pereira et al., 2008). Therefore, basic fuchsin staining cannot be considered unequivocal proof for the presence of lignin, but it reinforces the data reported in this study.

Taken together, these data support the general lignification of endosperm cell walls, with particular emphasis on the transfer cells despite previous contradictory reports (Gunning and Pate, 1974; Gaymann and Lörcher, 1990; Heide-Jørgensen and Kuijt, 1994; Vaughn et al., 2007). These discrepancies probably reflect the higher sensitivity of our staining methods compared to the periodic-Schiff reaction plus alcian blue or toluidine stains used by Gunning and Pate (1974), and the phloroglucinol stain used by Vaughn et al. (2007). We also tested phloroglucinol, and found as expected that this method did not detect any lignin in maize endosperm, only in the vascular tissue of the maize kernel (data not shown). Several authors have demonstrated that phloroglucinol is not sensitive enough for early lignification studies (Kutscha and McOrmond, 1972; Müsel et al., 1997).

The lack of significant differences in lignin content between ingrowths and adjacent walls after 6 DAP reinforces their similarity in composition (Vaughn et al., 2007). However, there seems to exist differences in lignin distribution at least in the reticulate ingrowths throughout development and differences in composition in the ingrowths and adjacent walls cannot be ruled out; this phenomenon must be addressed in future experiments.

Lignin is mainly associated with cell wall rigidity and strength, but it is also present in low amounts in the growing primary cell walls of several species (Joseleau and Ruel, 1997; Müsel et al., 1997; Christiernin et al., 2005; Gritsch and Murphy, 2005). We detected lignin in maize MBETCs at 6 DAP, an early developmental stage characterized by a high growth rate, allowing the cells to increase by up to 4-fold in area until 12 DAP (Table 1, Figure 3A). The ingrowths also form and develop extensively during this period (Figures 1, 2; Monjardino et al., 2013). The flange ingrowths may restrict cell expansion, because they are predominantly obliquely oriented in relation to the long axis of the cell, and are often as long as the cells themselves (Talbot et al., 2002; Monjardino et al., 2013). They also appear to be attached to the adjacent wall along most of their length and, as we demonstrate in this study, they are also lignified. Furthermore, the oblique orientation of the flange ingrowths does not facilitate cell expansion along the long axis, although this may not necessarily act as an impediment because the ingrowths may behave like the folding bellows of an accordion, expanding obliquely to their orientation. In addition, the increasing angle between flange ingrowths and the nearest anticlinal walls during development (Figure 3C) opposes the stretching effect of the predominant orientation of cell growth. Therefore, other factors may facilitate ingrowth reorientation in the MBETCs: (i) new ingrowths may be formed with greater angles; (ii) the distortion effect on the transfer cells caused by the growing embryo could promote unequal variations in ingrowth and anticlinal wall orientations; (iii) assimilate flow may contribute to distortions in the orientation of the ingrowths; and (iv) the connections between the ingrowths and walls may become weaker as the cell expands and develops, therefore enabling them to change their orientation.

The starchy cells also contain lignin throughout development (Tables 1, 3), despite the cells grew even more significantly than the transfer cells (Figure 3B). These cells accumulate large amounts of starch and protein, and at later developmental stages they lose most of their water content. Therefore, the walls of the starchy cells must be strong and flexible enough to endure such challenging conditions, and lignin is likely to be an important constituent that provides such abilities.

Our results suggest that the role of lignin in the structure of the cell wall should be reconsidered. Müsel et al. (1997) proposed that, in the process of cell wall growth, lignin may act to counterbalance independent wall-loosening processes (mediated by growth-promoting agents), thus allowing the cell wall to expand without losing rigidity. Our results support this hypothesis for maize transfer and starchy endosperm cells. The role of lignin in ingrowth formation must also be to stabilize its structure, as that seems particularly to be the case in the inner loose ends of the reticulate ingrowths.

### Conflict of interest statement

The authors declare that the research was conducted in the absence of any commercial or financial relationships that could be construed as a potential conflict of interest.
